# Facilitating interprofessional collaboration for effective care transitions of a patient with chronic obstructive pulmonary disease

**DOI:** 10.1002/ncp.70103

**Published:** 2026-02-23

**Authors:** Sara Edwards, Kaitlyn Kolcun, Jeanie Bochenek, Emily Buatois, Monica Robinson, Erin Thomas, Matthew Curtis, Catherine Hechmer, Tracy Taylor, Sheela Thomas

**Affiliations:** ^1^ College of Nursing The Ohio State University Columbus Ohio USA; ^2^ College of Pharmacy The Ohio State University Columbus Ohio USA; ^3^ School of Health and Rehabilitation Sciences, College of Medicine The Ohio State University Columbus Ohio USA; ^4^ Division of Respiratory The Ohio State University Wexner Medical Center Columbus Ohio USA; ^5^ College of Social Work The Ohio State University Columbus Ohio USA; ^6^ Division of Dietetics The Ohio State University Wexner Medical Center Columbus Ohio USA

**Keywords:** collaboration, communication, comorbidities, interprofessional collaboration (IPC), interprofessional team, transitions of care (TOC)

## Abstract

Transitions of care are the movement of a patient from one care setting or provider to another. Interprofessional collaboration is critical in ensuring patient safety and satisfactory health outcomes. Each time an interprofessional team transfers a patient, the team performs three important roles: representing the patient, providing patient information for other team members, and coordinating the transition. Poor transitions of care may contribute to negative health outcomes, especially for patients with chronic health conditions, complex medication regimens, and high‐risk treatments. We present a case study of a patient with complicated chronic obstructive pulmonary disease that depicts the importance of successful interprofessional collaboration during the transition of care from hospital to home illustrating the unique contributions of the various disciplines involved in the patient's care.

## INTRODUCTION

Managing patients with chronic health conditions can be difficult, as they often have competing comorbidities. Effective care for these patients necessitates coordinated interprofessional collaboration (IPC) to ensure comprehensive management as patients transition across care settings and ultimately to home. Transitions of care (TOCs) from one setting to another significantly increase the patient's risk for adverse events.^1^, ^2^, ^3^ To facilitate this understanding of how effective collaboration, communication, and coordination occurs within an IP team, a case study on a patient hospitalized with chronic obstructive pulmonary disease (COPD) scheduled for discharge to home will be discussed.

### IPC

IPC is an integral part of healthcare and critical in ensuring patient safety and satisfactory healthcare outcomes. When an IP team works together, they bring diverse expertise, perspectives, and specialized knowledge to address complex challenges throughout a patient's care experience.^4^, ^5^ This team‐based approach is especially important during TOCs, whether the patient is moving between different healthcare levels, healthcare facilities, or returning home. Effective communication is an essential part of IPC.^1^, ^5^ Miscommunication or conflict among the IP team can compromise patient care.^1^, ^3^ To achieve an effective TOC, strategies may include staff development programs, regular IP meetings, and the use of standardized communication tools or resources.^6^

### TOCs

TOCs are assessed using various metrics, such as the Hospital Consumer Assessment of Healthcare Providers and Systems, the Care Transitions Measure, hospital readmission rates, and adverse event rates. The LACE index is a validated tool that predicts readmission risk based on key factors: length of stay, acuity, comorbidities, and emergency visits.^7^, ^8^ Each factor is assessed, and the summative score is used by clinicians for discharge planning and postdischarge support. When poor TOC occurs, there may be an increased risk for negative healthcare outcomes for patients, such as adverse drug events.^8^ Adverse drug events are particularly prevalent for patient populations with complex medication regimens, high‐risk treatments, and older adults as they navigate healthcare systems.^8^ Ineffective TOC may occur when patients have limited language proficiency in the language used by healthcare providers, low health literacy, poor understanding or engagement with the care plan, conflicting provider recommendations, complex medication regimens, and unclear follow‐up instructions as well as the exclusion of patients and caregivers from the planning process.^8^ Patient and caregiver involvement can help identify and address anticipated needs, supporting a smoother TOC.^9^

### COPD: A complex health concern

There are many chronic conditions for which IPC is critical for successful transitions in healthcare settings. This discussion will focus on COPD, a complex and multifaceted disease. COPD, which includes chronic bronchitis and emphysema, is an irreversible disease that is the fourth leading cause of death worldwide and the sixth leading cause of death in the United States.^10^, ^11^, ^12^ After discharge to home, 20% of patients with COPD encounter some type of adverse experience such as injuries from care, drug reactions, infections, surgical complications, deterioration of symptoms, extended illness, or hospital readmission.^11^, ^13^ Many of these complications occur because of miscommunication or a lack of communication among the IP team regarding the patient's condition, plan of care, or follow‐up care needs.^13^ Effective IPC and communication are essential for coordinating care and reducing complications such as adverse events, which are associated with poor TOC.^1^, ^2^, ^3^, ^8^, ^9^

## CLINICAL CASE STUDY

This case example of a 64‐year‐old patient hospitalized with a hip fracture, exacerbation of COPD, and complex nutrition deficits represents a composite, hypothetical patient constructed from common clinical presentations. It does not reflect any specific individual.

The patient has no known allergies and a past medical history notable for COPD, hypertension, stage II supraglottic carcinoma status postpharyngectomy, and osteoporosis. They are preparing for discharge home after a 2‐week hospitalization for a right hip fracture with reconstructive orthopedic surgery and COPD exacerbation.

The patient had a gastrostomy tube in place for bolus enteral nutrition (EN) with minimal oral intake before admission secondary to a history of supraglottic cancer and pharyngectomy. The gastrostomy tube is also used for medication administration. The patient and their partner live independently at home and are comfortable with administering bolus feeds. The patient is a current smoker with a 60‐pack‐year smoking history but is making significant progress on cutting back. Support is available from an adult child and grandchild who live 1 h away. The adult child works full‐time and is only available to visit on weekends. The patient is concerned about remaining the caregiver for their partner, who recently experienced a myocardial infarction and requires assistance with activities of daily living (ADLs) and transportation to follow‐up appointments.

The patient presented with a right hip fracture and compromised respiratory status with shortness of breath and hypoxia secondary to COPD and was allowed nothing by mouth in anticipation of surgery the next day. Postoperatively, the patient was transferred to the intensive care unit (ICU) from the surgical unit because of worsening dyspnea on exertion and inability to maintain oxygen saturation. The ICU stay was complicated by postoperative ileus, which led to additional time on nothing by mouth status. Intravenous fluids (IVFs) including dextrose and potassium supplementation were administered owing to hypoglycemia and hypokalemia during interruption of EN and nothing by mouth status. During this time, the patient received 25% of their estimated energy requirements for 4 days.

Eventually, the patient stabilized with 2 L of oxygen via nasal cannula and was transferred to the surgical unit. On return of bowel function, continuous EN was initiated. As EN was advanced, IVFs were tapered and subsequently discontinued without complication. The patient was advanced to a pureed diet with nectar‐thick liquids with continued EN after bedside swallow evaluation by the speech‐language pathologist. The patient was cleared to eat a mechanical soft diet by mouth as tolerated. The patient exhibited ongoing COPD symptoms of low oxygen, shortness of breath, poor endurance, poor appetite, and limited oral intake (<50% of meals); EN was transitioned to bolus feeds for discharge. Bolus feeds were preferred by the patient because of their familiarity with this mode of nutrition support.

The patient had a significant weight loss of 3% during the 2‐week hospitalization, resulting in a body mass index of 18 kg/m^2^. Nutrition assessment revealed severe malnutrition when applying the Academy of Nutrition and Dietetics (AND) and American Society for Parenteral and Enteral Nutrition (ASPEN) criteria using the following parameters: <75% of estimated nutrition needs consumed for 1 month, 8% weight loss within 3 months, severe fat loss, and severe muscle loss.^14^ In obtaining the patient's preadmission history, the team discovered that the patient's EN bolus feeds were often missed because of their partner's illness.

On the day of discharge, the patient is alert and oriented to person, place, time, and situation, and their appearance correlates with stated age but met the criteria for cachexia.^11^, ^15^ The patient reports feeling fatigue throughout the day with feelings of depression and denies headaches and vision changes. The patient is quiet and appears to be withdrawn. The patient's skin is warm and dry. Assessment of the upper extremities shows no digital clubbing, pale digits, capillary refill >3 s, and pulse oximetry of 90% on 2 L of oxygen via nasal cannula. On assessment of respiratory effort, the patient has labored breathing with an irregular rate of 26, and bilateral lung auscultation revealed diminished breath sounds with inspiratory crackles. The patient's abdomen is soft, with bowel sounds present in all four quadrants. The patient's posture and alignment of the extremities and spine are symmetrical.

The assessment was that the patient will benefit from continued nutrition support, exercise training (pulmonary rehabilitation), and pharmacologic interventions.^15^ The discharge plan included bolus feeds of 365 ml of a standard polymeric formula four times per day and pureed diet with nectar‐thick liquids to meet the energy and protein requirements necessary to gain weight and improve muscle mass.^15^ The patient will be discharged home on oxygen to keep oxygen saturation between 88% and 92% and to minimize dyspnea.^16^ Discharge medications included blood pressure medication, anticoagulant therapy to prevent postoperative deep vein thrombosis, two inhalers for COPD symptom management, a short‐acting beta‐agonist and a short‐acting muscarinic antagonist, and multivitamin and vitamin D supplementation.

## COPD MANAGEMENT: AN IP HEALTHCARE PERSPECTIVE

This section will discuss the above patient through the professional lenses of the IP team members including the registered dietitian (RD), the respiratory therapist (RT), the pharmacist, the physical therapist (PT), the occupational therapist (OT), social worker (SW), and nurse.

### Nutrition profession

Malnutrition is common in COPD. A 2023 systematic review and meta‐analysis estimated a pooled malnutrition prevalence of approximately 30% (with ~50% at‐risk) and found malnutrition was associated with worse lung function, poorer exercise tolerance, and a greater mortality risk.^16^ In studies following groups of patients with COPD referred for pulmonary rehabilitation, malnutrition by Global Leadership Initiative on Malnutrition (GLIM) criteria was highly prevalent (~45%) and was associated with nearly three times greater mortality and higher hospitalization risk.^17^ Similarly, malnutrition defined by ASPEN criteria in rehabilitation cohorts predicted approximately fourfold increased long‐term mortality.^18^ Therefore, all patients with COPD should be screened for malnutrition using validated tools such as the GLIM or ASPEN criteria, with regular assessment of body weight, fat‐free mass, and dietary intake.^15^, ^17^

Multiple factors drive malnutrition in COPD, all of which are described in recent reviews. These factors include increased work of breathing and resting energy expenditure, systemic inflammation and catabolism, dyspnea and early satiety that reduce oral intake, frequent exacerbations and hospitalizations, polypharmacy, and social or functional barriers to eating.^18^, ^19^ Energy and protein requirements vary based on factors such as age, disease state, activity level, and body weight. Because low body weight and low fat‐free mass are independently associated with worse outcomes, the AND Evidence Analysis Library recommends individualized energy prescriptions and suggests using ~30 kcal/kg body weight as a starting estimate for nonobese adults with COPD with monitoring and adjustments as needed.²¹ For protein intake, practice guidelines and reviews commonly target ~1.0–1.2 g/kg/day (higher in some protocols for catabolic or older patients) to preserve lean mass and function.^19^, ^20^ According to a meta‐analysis, increasing energy intake to approximately 318 ± 157 kcal/day above baseline intake leads to statistically significant positive weight gain and improvements in both respiratory and nonrespiratory muscle mass, which independently correlates with better survival rates in nutritionally depleted patients with COPD.^15^, ^18^

At TOC, the nutritionist recommended this patient consume small, frequent, and nutrient‐dense meals with nutrition supplements as needed through oral intake or gastrostomy tube. The patient's weight, body composition, and functional status need to be monitored regularly with the nutrition plan adjusted accordingly.

Micronutrients, particularly vitamin D, should be assessed based on the AND recommendations of checking serum 25‐hydroxyvitamin D and supplementing if levels are ≤10 ng/ml (and considering supplementation for 11–29 ng/ml), as evidenced by a randomized control trial demonstrating a reduction of exacerbations in deficient patients when supplemented. Evidence for other single vitamins/minerals is mixed; however, combined antioxidant/micronutrient formulations have shown some benefit in trials.^21^, ^22^

### Respiratory profession

Before discharge, the RT coordinated with nursing to determine supplemental oxygen needs to maintain ordered oxygen saturation levels. The RT communicated the respiratory equipment needs and oxygen requirements to the SW team to arrange for home delivery before the patient's arrival. Patients with partial pressure of oxygen <55 mm Hg on room air should be prescribed supplemental oxygen to maintain saturation >90% and reassessed within 60–90 days after discharge.^16^ Comprehensive care should include patient and caregiver education on equipment safety and emergency procedures, especially with home oxygen use because of the fire risk.^3^

Instructions to the patient about proper medication delivery included a long‐acting muscarinic antagonist and long‐acting beta‐agonist combination inhaler and a short‐acting beta‐2 adrenergic agonist as needed for dyspnea or exercise intolerance. During TOC, the RT had the patient demonstrate and explain inhaler use to confirm the correct technique for proper medication delivery at home.

RT had several recommendations that were communicated to the IP team regarding the TOC. It will be important for the PT and OT to know the patient's use of supplemental oxygen. The RT communicated suggestions for optimizing oxygen therapy to address the current level of dyspnea. The RT recommended timing strenuous physical activity while home caregivers are present, which includes PT, OT, nursing, and the caregiver(s). The RT recommendation was for strenuous activities to coincide with the peak effects of bronchodilators to help in reducing dyspnea and improving the patient's overall tolerance, especially when it comes to light housework or going outside the home for doctor's appointments or shopping. The RT communicated to the IP team the recommendation that smoking cessation and emphasis on oxygen safety are needed to promote a healthier lifestyle. Other shared recommendations included education on pursed‐lip breathing techniques and assessing accessibility to “as needed” bronchodilators.

### Pharmacy profession

Pharmacists play a critical role in recommending a therapeutic medication regimen based on the routes of administration and ensure medication reconciliation during all TOC. They are essential for managing the added complexity of EN. They evaluate medication orders to ensure the correct route of administration (eg, oral vs gastrostomy tube) and assess formulation suitability for crushing or mixing. Extended‐release or enteric‐coated medications should never be crushed, and resources such as the Institute for Safe Mediation Practices “Do Not Crush” list can guide safe practices.^17^ Pharmacists advise on absorption issues related to enteral access sites and recommend alternatives when needed.

To prevent complications, medications should not be administered simultaneously with EN. Pharmacists educated the staff and caregivers on holding EN during medication administration, flushing the tube before and after each dose, and coordinating with dietitians to minimize interruptions in nutrition.^17^ They can also help troubleshoot clogged enteral access devices, which occur in 23%–35% of patients.^21^

For this patient who currently uses two short‐acting inhalers, persisting COPD symptoms warrant a long‐acting inhaler.^16^ Because of postpharyngectomy anatomy, a metered‐dose inhaler (MDI) with a spacer was preferred over a dry powder inhaler. A long‐acting muscarinic antagonist and long‐acting beta‐agonist combination inhaler were recommended for daily maintenance therapy. Pharmacists are equipped to facilitate smooth transitions for all aspects of a patient's medication therapy regimen across all care settings.

### Physical therapy and occupational therapy professions

Physical therapy plays a significant role in transitioning patients with COPD from the hospital to home by focusing on restoring mobility, improving exercise tolerance, and reducing dyspnea through targeted interventions.^20^ Effective discharge planning relies on strong IPC between the PT and other members of the IP team to comprehensively address both functional endurance and mobility. Physical therapy coordinates closely with the RT to align breathing strategies with physical activity, ensuring patients maintain safe oxygen saturation levels during exercise.^22^ It was important for the PT to communicate with the RT about the patient's readiness for increased exercise levels. A key component of the exercise prescription is resistive exercises, particularly targeting quadriceps strength to improve walking distance, which has been shown to reduce reports of breathlessness.^23^

Before discharge, the PT communicated to the IP team about inspiratory muscle training that the patient received to help improve breathlessness.^24^ To avoid patient fatigue, collaboration and coordination of care among the PT, OT, and nurses can ensure that symptom monitoring and oxygen titration are integrated into the patient's mobility and self‐care activities.^20^ Because this case study patient is also post–hip fracture reconstruction, communication to the IP team related to balance and gait impairments should promote safe mobility not only in the hospital but also following discharge home. Physical therapy collaborated with nursing and the OT to ensure appropriate weight‐bearing precautions and mobility assistance, such as the use of a rolling walker and proper guarding against falls.

Occupational therapy is essential in preparing the patient diagnosed with COPD for increased independence at home, which requires collaboration with the IP team to ensure a comprehensive, holistic approach. Occupational therapy works with speech‐language pathology (SLP) and the RD to help the patient integrate feeding strategies and energy needs into daily routines, which are critical to preventing nutrition depletion in COPD. As part of TOC, the OT collaborated with SLP to address dysphagia concerns and recommended implementing safe swallowing. Occupational therapy plays a crucial role in helping patients with COPD manage breathing and eating coordination to regulate airflow and reduce breathlessness during meals, ensuring patients can pace their breathing while chewing and swallowing.^25^ When working with the patient, the OT can recommend positioning strategies to nursing, such as upright posture and forward‐leaning recovery positions, to optimize respiratory function while eating and during other activities.

Both the OT and PT should communicate closely with pulmonology and the RT to ensure the patient understands oxygen therapy needs related to activity, proper breathing techniques to reduce fatigue and improve endurance, and consistent use of incentive spirometry and/or other positive expiratory pressure therapy devices. The PT and OT communicated to nursing the importance of obtaining baseline vital signs before activity. Self‐management education, such as the effective use of inhalers, recognizing exacerbation signs, and implementing individual action plans should be integrated across all disciplines. This collaboration ensures consistency and carryover at discharge and prepares patients to handle their care at home.^25^ Providing the RD with detailed information about activity levels and responses to exercise interventions such as fatigue, delayed recovery, or lack of strength improvement are essential contextual factors for the RD to accurately estimate energy needs based on energy expenditure. Additionally, given the patient's withdrawn affect, the PT and OT should closely monitor this presentation and maintain ongoing communication with the IP team regarding any improvement or deterioration in response to the medical team's recommended implemented interventions. Finally, coordination and communication with the SW for equipment and therapy needs for mobility and self‐care activities at home are essential to ensure a safe TOC.

### Social work profession

The social work team plays a pivotal role in navigating a successful transition home, so patients and families have the support that ensures they can recover, be as independent as possible, and prevent adverse events and hospital readmissions. In assessing patients for discharge readiness, the SW finds that some patients and families need a higher level of care than what can be provided at home. This realization poses challenges for patients. When the SW determines these gaps, the patient may need to recover at a rehabilitation or long‐term care center rather than at home.

Concerning the patient in the case study, the SW ensured the patient understood the discharge plans for medications, nutrition intake, therapies, and follow‐up appointments. The SW needed to assess the needs of the patient and communicate with the IP team to discover what discharge equipment is needed at home.^26^ For instance, a patient with COPD may require a front‐wheeled rolling walker, a raised toilet seat, and hand‐reaching devices. The SW team also arranged for referrals for home health, OT and PT rehabilitation, patient and caregiver(s) education and training before discharge, and home equipment and supplies delivery.^26^ The SW team can connect the patient with local community resources that provide home delivery of medications, supplies, groceries, meals, and even cleaning services.

Given that the patient has COPD, mobility and increased work of breathing can be barriers to adequate nutrition, so home delivery services can help the patient be independent.^26^ The SW can also help the patient and their partner find transportation to follow‐up medical appointments. The patient's disease acuity, along with the IP healthcare team's observations that the patient was quiet and withdrawn and experiencing a lack of appetite, energy, and motivation led the SW to recommend consultation with psychiatric services for evaluation for depression before discharge. The SW communicated to the IP team that these symptoms are particularly dangerous for this patient because of weight loss, challenges obtaining adequate nutrition during the hospital stay, and limited ability to engage successfully in ongoing therapies and recovery.

### Nursing profession

When patients transition home, nurses are often the last IP team member to coordinate their care and identify readiness for discharge. During collaboration, nursing recommended identifying home health services, resources within the community, transportation needs, and other gaps limiting continuity of care.

Nursing partnered with the PT/OT to assess the patient's functional needs, including ADLs, mobility, and cognitive function. To help prevent future respiratory exacerbations and hypertensive episodes, nursing worked closely with the RT, RD, and pharmacy to develop an individualized care plan that optimized timing for medication, nutrition, and respiratory treatments. Nurses coordinated with the IP team to ensure that all medical equipment and supplies are delivered and set up at home for the patient to provide a smooth TOC. Nursing ensured referrals, prior authorizations, and follow‐up visits for primary care, pulmonology and pulmonary rehabilitation, and the PT/OT. Additionally, collaboration with the pharmacy included medication reconciliation and patient education to reinforce adherence to the medication regimen. Nursing emphasized to the IP team the need for the patient to have appropriate vaccinations to prevent infections that could exacerbate and further complicate COPD symptoms.^27^, ^28^, ^29^ A final recommendation to the IP team was to offer smoking cessation to improve health outcomes.^16^, ^30^

During the TOC, nursing communicated to the patient signs and symptoms of COPD exacerbation and when to seek medical care such as changes in sputum color and volume, dyspnea, cough, signs of hypercapnia, fatigue, and coronavirus disease 2019 symptoms.^16^ A recommendation was to avoid ongoing environmental exposure to potential irritants such as fumes, gases, dust, and smoke.^16^ Additionally, nursing recommended minimizing extreme temperature exposures, as this can increase the risk of exacerbation.^16^

## DISCUSSION

### IPC during TOCs

TOC requires coordinated efforts among multiple disciplines across healthcare settings. IPC is integral to mitigating risk associated with medication errors, inconsistent and insufficient patient education, and adverse events along with fragmented care. IPC provides a collaborative framework creating structured communication and shared accountability. For this patient example, each member of the IP team shared their considerations for the patient's discharge through their professional lens, underscoring the critical role of IPC in providing safe and effective patient‐centered care, particularly during TOC. Each profession involved within the IP team contributed unique expertise in anticipating patient needs, identifying gaps, securing necessary resources, and preventing fragmented or inconsistent care. Seamless coordination and communication among the IP team promotes a comprehensive and effective discharge plan that reduces the risk for postdischarge complications, hospital readmission, and adverse events.^20^

### Medication safety and education

Because of the complexity of COPD, the patient presented in the case study had a significant risk for hospital readmission if they encountered an inadequate IPC throughout the TOC. TOCs are high‐risk periods for patients with COPD, during which 66%–72% of medication errors occur.^1^ Effective TOC requires thorough medication reconciliation to ensure accurate medication lists, patient education on proper timing and administration, and resources to access the medication.^31^, ^32^ Pharmacist involvement reduces 30‐day readmission rates, as demonstrated by Mekonnen et al in a meta‐analysis reporting a 19% reduction in all‐cause readmissions in adult patients when pharmacists conducted discharge medication reconciliation.^33^ Postdischarge follow‐up, such as phone calls within 2–4 days, further supports continuity of care. These interventions are most effective when implemented collaboratively.^34^

Effective management of patient care relies on robust IPC and patient education. Education on proper inhaler use, particularly MDIs, should involve the RT, nursing, and pharmacy to prevent adverse events and reduce readmissions. Routine assessment of inhaler technique, access to medications, and adherence is critical and can be supported by the SW during the TOC.^14^ Nonpharmacological therapies, including positive expiratory pressure devices and breathing techniques, require regular evaluation by the RT and PT. Pulmonary rehabilitation programs coordinated by the IP team can improve symptoms and reduce anxiety in patients with COPD.^14^

## CONCLUSION

IPC is essential in ensuring a successful TOC and achieving optimal health outcomes for patients. Effective IPC requires that each IP team member demonstrate mutual respect and recognize the unique and valuable contributions that each discipline plays in the delivery of safe patient‐centered care. IPC enhances the effectiveness of healthcare professionals allowing for a smoother TOC and limiting the risk for errors and adverse events.^35^, ^36^, ^37^, ^38^

## AUTHOR CONTRIBUTIONS

Sara Edwards contributed to the conceptualization (lead), writing—original draft (lead), writing—review and editing (lead), and supervision (lead). Kaitlyn Kolcun contributed to the writing—original draft and writing—review and editing (equal). Jeanie Bochenek contributed to the writing—origianl draft (equal) and writing—reviewing and editing (equal). Emily Buatois contributed to the writing—original draft (equal) and writing—reviewing and editing (equal). Monica Robinson contributed to the writing—orginal draft (equal) and writing—reviewing and editing (equal). Erin Thomas contributed to the writing—original draft and writing—review and editing (equal). Matthew Curtis contributed to the writing—origianl draft (equal) and writing—reviewing and editing (equal). Catherine Hechmer contributed to the writing—original draft (equal). Tracy Taylor contributed to the writing—original draft (equal) and writing—reviewing and editing. Sheela Thomas contributed to the writing—original draft and writing—review and editing (equal).

## CONFLICT OF INTEREST STATEMENT

None declared.
